# Cardiac arrest in a young adult with complex congenital heart disease: an echocardiographic diagnostic challenge in the acute setting

**DOI:** 10.1093/ehjimp/qyaf045

**Published:** 2025-04-16

**Authors:** Floran Sahiti, Fabian Kerwagen, Stefan Frantz, Dominic Schröder, Dirk Weismann

**Affiliations:** Department of Medicine I, University and University Hospital Wurzburg, Oberdürrbacher Str. 6, 97080 Wurzburg, Germany; Department of Clinical Research and Epidemiology, Comprehensive Heart Failure Center, University and University Hospital Wurzburg, Am Schwarzenberg 15, Haus A15, 97078 Würzburg, Germany; Department of Medicine I, University and University Hospital Wurzburg, Oberdürrbacher Str. 6, 97080 Wurzburg, Germany; Department of Clinical Research and Epidemiology, Comprehensive Heart Failure Center, University and University Hospital Wurzburg, Am Schwarzenberg 15, Haus A15, 97078 Würzburg, Germany; Department of Medicine I, University and University Hospital Wurzburg, Oberdürrbacher Str. 6, 97080 Wurzburg, Germany; Department of Pediatrics, University and University Hospital Würzburg, Würzburg, Germany; Department of Medicine I, University and University Hospital Wurzburg, Oberdürrbacher Str. 6, 97080 Wurzburg, Germany

**Keywords:** congenital heart disease, Fontan physiology, echocardiography, intensive care unit, arrhythmia

We report the case of a young adult in their early 20s admitted to the intensive care unit (ICU) following successful pre-hospital resuscitation for ventricular fibrillation. The patient was reportedly in good health but had a history of complex congenital heart disease. Initial ventilation was provided via a laryngeal tube, followed by endotracheal intubation upon ICU admission. A standard post-cardiac arrest workup was performed. Detailed evaluation of the medical history revealed heterotaxy syndrome with an atrioventricular canal defect, transposition of the great arteries, severe pulmonary stenosis, hypoplastic pulmonary system, and persistent ductus arteriosus. The patient initially underwent a Glenn anastomosis at the age of 2, followed by a Fontan completion with an extracardiac conduit and a fenestration at the age of 3. A paediatric cardiologist was promptly involved to assist with the assessment.

The electrocardiogram showed no abnormalities. Echocardiographic assessment in such cases is particularly challenging due to the complex and non-intuitive anatomy. Key priorities include evaluating ventricular function and ruling out causes of obstructive shock, i.e. pulmonary embolism. Other differential diagnoses include Fontan circuit failure as well as electrolyte and metabolic abnormalities. These patients are at increased risk of developing arrhythmias and advanced heart failure due to persistently elevated central venous pressure, reduced preload to the systemic ventricle, and impaired cardiac output augmentation during stress or exercise.

Echocardiography revealed a large atrioventricular canal defect with systemic circulation supported solely by a functional single ventricle, with a left ventricular dominance and a reduced systolic function with ejection fraction of 30% and TAPSE of 7 mm. Additionally, there was atrioventricular valve insufficiency with multiple jets (Grades II–III), but no significant enlargement of the common atrium (*Figure 1A* and *B*). The normal respiratory-dependent flow acceleration, indicating unobstructed pulmonary perfusion (*Figure 1C* and *D*), suggested that overall, arrhythmia was the most likely explanation. The low-pressure Fontan circulation is difficult to visualize echocardiographically, with imaging limited to the superior and inferior vena cava (*Figure 1C* and *D*) and, occasionally, the lower part of the extracardiac conduit.

**Figure 1:**
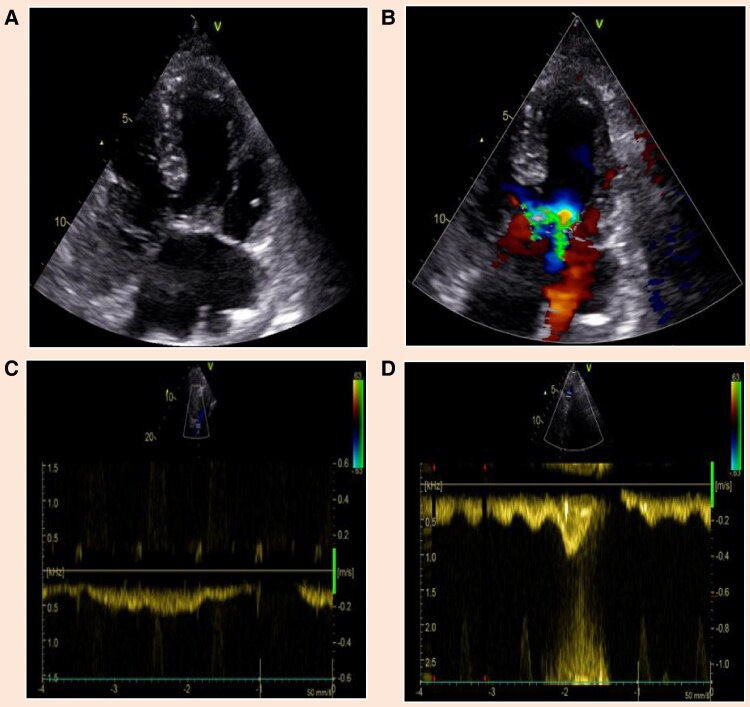
(*A* and *B*) A large atrioventricular canal defect, with systemic circulation solely supported by a functional single ventricle exhibiting a reduced ejection fraction (30%) and TAPSE of 7 mm as well as atrioventricular valve insufficiency (Grades II–III) with multiple jets (see Supplementary data online, *Videos S1* and *S2* as well). No significant enlargement of the common atrium was observed. (*C*) The transition between the inferior vena cava and the Fontan tunnel, showing a respiratory-modulated flow velocity of 0.2–0.3 m/s. (*D*) The upper segment of the superior vena cava, with a respiratory-modulated flow velocity of up to 0.4 m/s. The transition into the pulmonary vessels was not visualized.

To optimize haemodynamics, invasive ventilation with low positive end-expiratory pressure (PEEP) (≤5) was chosen to support passive lung perfusion and catecholamine therapy was required. Since our hospital is not a certified centre for adults with congenital heart disease, the patient was transferred within 12 h to the specialized centre, where the patient was treated before.

Despite improved survival following surgical palliation, adverse events remain common, with many patients experiencing a major event within 20 years of follow-up. While echocardiography remains valuable at the bedside, imaging modalities such as computed tomography angiography (CT-A) and cardiac magnetic resonance imaging (cMRI) play a crucial role in definitively ruling out intracardiac thrombi or obstructions within the Fontan circuit. A distinctive aspect of these patients is that the low-pressure Fontan circulation is typically not assessable via echocardiography in adults. Only the superior and inferior vena cava, and in some cases, the lower portion of the extracardiac conduit, can be visualized in subxiphoidal view. A respiratory-dependent flow acceleration may suggest unobstructed pulmonary perfusion, as observed in this patient, further supporting the exclusion of a pulmonary embolism.

This case highlights the complexity of diagnosing and managing acute events in adult patients with congenital heart defects, particularly those with Fontan physiology. Effective care in critical settings requires expertise in adult congenital heart disease and a tailored diagnostic and therapeutic approach.

## Supplementary data

Supplementary data are available at *European Heart Journal - Imaging Methods and Practice* online.

**Consent:** Written consent was obtained from the patient's family.

**Funding:** None.

**Data availability:** Anonymized information (echocardiography images) will be shared upon reasonable request to the corresponding author.

## Lead author biography


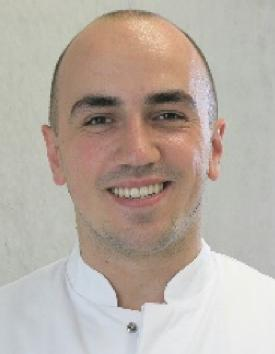


Floran Sahiti is a cardiology resident in the Department of Internal Medicine I at the University Hospital Würzburg, Germany, and a research associate at the Comprehensive Heart Failure Center in Würzburg. He completed his medical degree at the University of Prishtina in the Republic of Kosovo and later earned his PhD at the Julius-Maximilians University Würzburg, Germany. Floran's primary interests include heart failure, cardiomyopathies, and advanced cardiac imaging studies.

## Supplementary Material

qyaf045_Supplementary_Data

